# Behavioral Phenotype, Electroclinical Features, and Treatment Options in Twins with *Lrp2* Candidate Variants (Donnay–Barrow/Foar Syndrome)

**DOI:** 10.1155/2023/6679572

**Published:** 2023-09-30

**Authors:** Alessia Mingarelli, Giovanni Battista Pipitone, Giacomo Torini, Maria Grazia Patricelli, Martina Totaro, Clara Colonna, Paola Carrera, Federico Raviglione

**Affiliations:** ^1^Hospital Neuropsychiatry Service, ASST Rhodense, Rho, Milan, Italy; ^2^Laboratory of Molecular Genetics, Cytogenetics and Clinical Genetics, IRCCS San Raffaele Scientific Institute, Milan, Italy; ^3^Unit of Genomics for Diagnosis of Human Disease, IRCCS San Raffaele Scientific Institute, Milan, Italy

## Abstract

The *LRP2* gene encodes megalin (LRP-2/GP330), a large single-spanning transmembrane glycoprotein that serves as a multiligand endocytotic receptor and mediates the reabsorption of albumin in the proximal renal tubule. *LRP2* is implicated in an autosomal recessive disorder characterized by dimorphisms, ocular anomalies, sensorineural deafness, proteinuria, epilepsy, and intellectual disability: a clinical condition called Donnai–Barrow syndrome (DBS) or facio-oculo-acoustico-renal (FOAR) syndrome. Pathogenic variants in *LRP2* have been reported in fewer than 60 patients, but a detailed description of seizures, electroencephalographic patterns, imaging findings, behavioral phenotype, and long-term follow-up is still needed. We provide a clinical report of two mono-chorionic twins with *LRP2*-related disease manifesting developmental delay, autistic features, seizures, proteinuria, and sleep disorders. By sequencing clinical exome, *LRP2* candidate rare variants, c.6815G > A, p. (Arg2272His), inherited from the mother and c.12725A > G, p. (Asp4242Gly), inherited from the father, were identified. During follow-up, at the age of 7, the main clinical features of the patients included insomnia, autistic features, severe psychomotor delay, and absent speech. The patients were under treatment with risperidone, antiseizure medications (ASMs), and supplementation of alpha-lactalbumin for self-injury and sleep disturbance. Our study confirmed the wide spectrum of behavioral and neurological and psychiatric features of this rare condition, suggesting new treatment options.

## 1. Introduction

Donnai–Barrow syndrome (DBS; OMIM 222448) is a rare autosomal recessive disorder first described in 1993 [1] Causative variants in the gene *LRP2* encoding low-density lipoprotein receptor-related protein 2 and located on chromosome 2q23.3–31.1 are responsible for the DBS/FOAR syndrome identified in all of the patients [2]. DBS and facio-oculo-acoustico-renal (FOAR) syndrome (OMIM 227920) share many clinical aspects [3], and, since 1993, fewer than 60 affected individual have been reported up to date [1, 3–7]. The LRP2 gene encodes megalin (LRP-2/GP330), a large 600 kda single-spanning transmembrane glycoprotein that comprises 4655 amino acids and serves as a multiligand endocytotic receptor, mediating the reabsorption of albumin in the proximal renal tubule through a process of endocitosys [8]. Megalin also binds more than 50 ligands, including lipoproteins, vitamin-binding proteins, hormones, enzymes, and immune and stress-response-related proteins. Decreased megalin expression appears to be responsible for the proteinuria observed in these patients. Megalin is also highly expressed on the apical surface of absorptive epithelia and is involved in embryonic development, important during forebrain development and developing renal proximal tube. It is also expressed in the lung, eye, intestine, uterus, oviduct, and male reproductive tract [8]. Apart from proteinuria, characteristic features of these patients include congenital anomalies (>90%) with typical craniofacial features (ocular hypertelorism, enlarged fontanelle, partial or complete agenesis of the corpus callosum, high myopia, retinal detachment, progressive vision loss, and iris coloboma), sensorineural hearing loss, and intellectual disability [9, 10]. Additional features such as congenital diaphragmatic hernia and/or omphalocele and macrocephaly are also reported [2]. Epilepsy has been described but remains a rare feature of the syndrome [4]. Intellectual disabilities of varying degrees ranging from mild to severe and is present in 87% of patients [2]. Other neuropsychiatric characteristics include delayed motor development, autistic features with Self-Injury Behaviors (SIBs) and sleep disorders [11]. Both inter- and intrafamilial phenotypic variability were observed. However, the neuropsychiatric phenotype is still poorly described, and only for few reported cases, we have a clinical follow-up.

We report the clinical features of two mono-chorionic twins with *LRP2*-related disease manifesting developmental delay, autistic features, seizures, proteinuria, and sleep disorders. Our aim, according to the current literature, is to better delineate and expand the phenotypic spectrum (electro-clinical and behavioral phenotype) of LRP2-related syndrome, identifying novel diagnostic indicators for tailored therapeutic options.

## 2. Case Report

Two 9-year-old female twins (patient 1 P.A. and patient 2 P.S.) were born from healthy unrelated Caucasian parents with intra-cytoplasmic sperm injection (ICSI). A mono-chorionic twin pregnancy resulted in an emergency delivery by cesarean section at 32 weeks of gestational age due to maternal preeclampsia. Both twins had transient respiratory distress. Apgar score was 5 (1 min) and 7 (5 min) for both twins without need of resuscitation. Pediatric and neurologic evaluations were performed for prematurity screening. Based on postnatal clinical examination and imaging studies, they were noted to have marked hypertelorism and a short nose with a broad tip. Patients were followed with a multidisciplinary regular follow-up (see clinical features in Table 1) that included neurological evaluation, neuroimaging studies, neurophysiological studies, ophthalmology evaluations, clinical examinations, laboratory testing, and genetic counseling. All the exams did not show any alteration except for isolated mixed and low molecular weight proteinuria presenting by alpha and beta 2-microglobulin, haptoglobin, and albumin in a 24 h urine sample, without renal failure. A genetic family study by Next Generation Sequencing (NGS) of clinical exome (about 4800 genes, TruSight One panel, Illumina) was performed.

### 2.1. Genetic Study

After sequencing the clinical exome of the parents and the daughters, variants with a MAF > 1%, noncoding deep-intronic were filtered out. On the remaining variants, those in agreement with an autosomal recessive hypothesis were prioritized. In a parallel analysis, after selection of variants with a MAF < 1%, a prioritization was performed with the tool Exomiser based on the following HPO descriptors: prominent ear helix (HP: 0009904); poor eye contact (HP: 0000817); abnormality of the forehead (HP: 0000290), fine hair (HP: 0002213); hypertelorism (HP: 0000316); aphasia (HP: 0002381); stereotypy (HP: 0000733); and global developmental delay (HP: 0001263). In both the procedures, *LRP2* variants were prioritized in the two probands: the NM_004525: c.12725A > G (rs35942532), causing the missense p. (Asp4242Gly), inherited from the father, and the NM_004525: c.6815G > A, not reported in dbSNP and causing the missense p. (Arg2272His), inherited from the mother. Both the variants were located within the class B-LDLR repeat protein extracellular domain and predicted in silico as possibly damaging. By applying ACMG criteria, both the variants have been classified as variants of unknown significance (VUS, class 3) [12]. Although mild clinic signs were present in our probands, regarding the absence of major organic manifestations (renal and ocular impairment), in contrast with the typical neurological and neurodevelopmental spectrum reported, it could be argued that a partial inactivation of the protein, due to the presence of a hypomorphic variant, may explain the observed phenotype.

### 2.2. Imaging Findings

**Figure 1:**
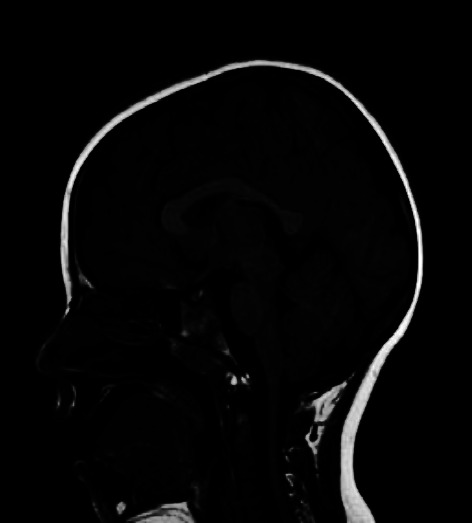
MRI sagittal seq. (5 yrs): P.2 showed a mild thickening of CC, an mild herniation of right cerebellar tonsil.

Both twins underwent neuro-radiological examinations. Brain MRI was performed at the age of 2 years and repeated at the age of 5. No major malformation of cortical development was noticed, but a mild thickening of the corpus callosum was described in both patients. In patient 2, a mild herniation of the right cerebellar tonsil was described (Figure 1).

### 2.3. EEG Findings

**Figure 2:**
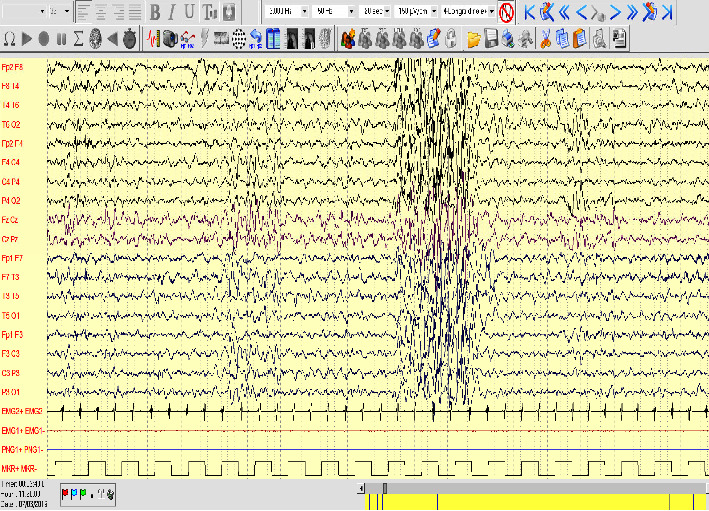
In P.2. the EEG recording showed a mild slowing of background activity with irregular and generalized spike and waves complexes during NREM sleep and drowsiness.

The patients underwent electro-clinical follow-up with serial Video-EEG sleep-poligraphic recordings. In both patients, recordings showed a slowing of background activity in the awake state and an atypical electroclinical pattern in sleep with sequences of theta monomorphic discharges over the anterior derivations. The epileptic patient (Pt.2) also presented with generalized spike-waves discharges (Figure 2).

### 2.4. Neuropsychiatric Features and Follow-Up

Patient 1 (P.A.) presented a severe developmental delay, with absence of speech and autistic features. Behavioral disorders (autistic SIB, psychomotor agitation and motor stereotypies) were the main features and required pharmacological treatment with risperidone. No sleep disorders or feeding problems were reported. patient 1 never experienced seizures.

Patient 2 (P.S.) presented a severe developmental delay with absence of speech and autistic features. Behavior disorders (autistic SIB, psychomotor agitation, and motor stereotypies) were the main features, more severe than in patient 1. Pharmacological treatment with Risperidone was required. She also presented epileptic seizures characterized by generalized tonic-clonic seizures during fever by the age of 2 years and afebrile seizures characterized by behavioral arrest, tonic upper limbs abduction, starting from the age of 4 years old. Treatment with valproic acid (500 mg/daily) was started with good control of seizures but interrupted due to collateral effects (marked weight gain). The drug was replaced by low dosage of topiramate (1.5 mg/kg/daily) with good control of seizures. Patient 2 also presented with a sleep disorder, characterized by frequent night awakening, reduced time of sleeping, and sleep-wake cycle inversion. Low-dosage treatment with mirtazapine (7.5 mg/daily) was started after 4 weeks of treatment with melatonine RP 2 mg/daily. Both twins manifested SIB as head banging, self-biting, and eye poking with a great irritability manifestation during all the night and day. For patient 2, emotional dysphoric dysregulation was also evident. Both twins recently underwent nephrological assessment that noticed mild increased albumin and total proteins level renal excretion.

At the age of 7 years, patients started treatment with alpha-lactalbumin (Serplus Complex®), and after two weeks of treatment, they presented, especially P.2, a great response on behavior, reduction of self-injury actions, and also a more regular sleep-wake cycle. Mirtazapine was tapered off, and neuroleptic treatment decreased. The effect was maintained throughout the entire period of treatment (about 6 months of follow-up).

## 3. Discussion

DBS is a very rare condition. No population-based incidence or prevalence data are available. About 60 individuals with clinical and/or molecular data with a diagnosis of DBS have been reported in the medical literature. Many of these individuals are members of a few consanguineous families, and no ethnic group predominates. No genotype-phenotype correlations are known to date, and the observed DBS-marked clinical variability may be probably influenced by an underlying genetic background. In our patients, the presence of two missense *LRP2* variants in compound heterozygosity, with a recessive mode of transmission, was in agreement with the clinical evidence; nevertheless, the two genetic variants have been classified as variants of unknown significance (VUS), and functional studies would be needed to confirm their pathogenic role. Previous descriptions of DBS about phenotypic heterogeneity revealed the following characterizations: diaphragmatic hernia, exomphalos, hypertelorism, agenesis of the corpus callosum, sensorineural deafness, and myopia [1, 2, 4].

Developmental delay, intellectual disability, and behavioral autistic features have been reported. Motor milestones are only slightly delayed, and most children become continents. No systematic studies of intellectual functioning exist, but available data suggest that all individuals with DBS have intellectual disabilities of varying degrees, ranging from mild to moderate. Often, formal assessment is difficult because of the severe vision and hearing deficits.

Epilepsy has been described but remains a rare feature [4]. The few data available from case reports [9] show that tonic-clonic seizures are the main seizure type starting in childhood or early adolescence. In one individual, the first episode of seizures developed into status epilepticus, leading to her demise. No single ASM has been demonstrated to be the treatment of choice specifically for this disorder, and therapy should follow standard guidelines. Our patients confirm these clinical evidence and we used standard ASMs for achieving seizure freedom.

Regarding behavioral and developmental aspects, to date, there have been few documentations of DBS being associated with the occurrence of SIB, autistic features, sleep disorder, and emotional dysregulation, though this behavior is relatively common among individuals with other genetic conditions and developmental delays. Roane et al. [11] described a child aged five years engaging in SIB that was responsive to behavioral therapy. Usually, SIB results as a consequence of other medical complications (i.e., detached retina and blindness). Anyway, the occurrence of SIB, as well as a detailed description of epilepsy, and seizures have not been documented within DBS, due in part to the relative lack of developmental information and lack of follow-up. Various drugs (i.e., fluoxetine, risperidone, lorazepam, and clonidine) have been prescribed to address similar challenging behavior but, from literature data, we know they were unsuccessful. Our patients showed head banging, self-biting, sleep disorder, and eye poking without any other specific organic disease that could explain the behavior. The administration of aripiprazole, risperidone, valproic acid, useful both for seizures and emotional instability, mirtazapine, and benzodiazepines did not allow a significant improvement. In consideration of the failure of neuroleptic and available antidepressant drugs, also often discharged for the significant collateral side effects (hepatic metabolism and renal excretion), we administered a nutraceutical product, alpha-lactalbumin (ALAC), with proven and documented efficacy for sleep disturbances, autism spectrum, and emotional dysregulation. We started ALAC supplementation at the age of 7 years in both patients, at a dosage of 1000 mg/daily by oral intake in the morning. Since after two weeks of treatment, they presented a great response on behavior, reduction of self-injury actions, and also a more regular sleep-wake cycle. Antidepressant drugs were subsequently tapered off, and it could be possible to decrease neuroleptic treatment. The efficacy on behavior was maintained during all the period of treatment (about 6 months of follow-up). Since heterogeneous LRP2 mutations have been associated with varying degrees of behavioral alteration, our experience supports the possibility of considering nutraceutical treatment in combination with comproved drugs.

## 4. Conclusion

We report a possible diagnosis of DBS/FOARS in twin sisters presenting isolated low molecular weight proteinuria in the absence of pronounced renal manifestations with consistent neuropsychiatric features and epilepsy. Despite the absence of the main organic manifestation and a well-controlled epilepsy phenotype, we found it very challenging to control autistic feature, sleep disorder, and behavior dysregulation with the failure of “classic” neuroleptic drugs and a surprising response to nutraceutical supplementation.

Our observation is in agreement with previous reports indicating the association of different *LRP2* variants with varying clinical pictures, confirming that the correlation between pheno- and genotype remains to be further studied in DBS/FOARS.

## Figures and Tables

**Table 1:** Characteristic clinical features.

	Patient 1 (P.A.)	Patient 2 (P.S.)
Sex	F	F
Hypertelorism	++	++
Downslanting palpebral fissures	+	+
High myopia	−	−
Iris coloboma	−	−
Short nose	+	+
Posterior angulated ears	−	−
Sensorineural deafness	−	−
Diaphragmatic hernia	−	−
Exomphalos/cord hernia	−	−
Malrotation of bowel	−	−
Agenesis corpus callosum	+ (partial)	+ (partial)
Developmental delay	++	++
Autistic features	++	++
Seizures	−	++
Sleep disorder	−	++
Proteinuria	−/+	+

## Data Availability

The data that support the findings of this study are available upon request from the corresponding author. The data are not publicly available due to privacy or ethical restrictions.

## References

[1] Donnai D., Barrow M. (1993). Diaphragmatic hernia, exomphalos, absent corpus callosum, hypertelorism, myopia, and sensorineural deafness: a newly recognized autosomal recessive disorder?. *American Journal of Medical Genetics*.

[2] Pober B. R., Longoni M., Noonan K. M. (2009). A review of Donnai-Barrow and facio-oculo-acoustico-renal (DB/FOAR) syndrome: clinical features and differential diagnosis. *Birth Defects Research Part A: Clinical and Molecular Teratology*.

[3] Kantarci S., Al-Gazali L., Hill R. S. (2007). Mutations in LRP2, which encodes the multiligand receptor megalin, cause Donnai-Barrow and facio-oculo-acoustico-renal syndromes. *Nature Genetics*.

[4] Chassaing N., Lacombe D., Carles D., Calvas P., Saura R., Bieth E. (2003). Donnai-Barrow syndrome: four additional patients. *American Journal of Medical Genetics*.

[5] Kantarci S., Ragge N. K., Thomas N. S. (2008). Donnai-Barrow syndrome (DBS/FOAR) in a child with a homozygous LRP2 mutation due to complete chromosome 2 paternal isodisomy. *American Journal of Medical Genetics, Part A*.

[6] Khalifa O., Al-Sahlawi Z., Imtiaz F. (2015). Variable expression pattern in Donnai-Barrow syndrome: report of two novel LRP2 mutations and review of the literature. *European Journal of Medical Genetics*.

[7] Patel N., Hejkal T., Katz A., Margalit E. (2007). Ocular manifestations of Donnai-Barrow syndrome. *Journal of Child Neurology*.

[8] Fisher C. E., Howie S. E. (2006). The role of megalin (LRP-2/Gp330) during development. *Developmental Biology*.

[9] Longoni M., Kantarci S., Donnai D., Pober B. (2018). *Donnai-Barrow Syndrome*.

[10] Veth K. N., Willer J. R., Collery R. F. (2011). Mutations in zebrafish lrp2 result in adult-onset ocular pathogenesis that models myopia and other risk factors for glaucoma. *PLoS Genetics*.

[11] Roane H., Bouxsein K., Fulton C. (2012). Assessment and treatment of self-injurious behavior associated with donnai-barrow syndrome. *Journal of Developmental and Physical Disabilities*.

[12] Richards S., Aziz N., Bale S. (2015). Standards and guidelines for the interpretation of sequence variants: a joint consensus recommendation of the American college of medical genetics and genomics and the association for molecular pathology. *Genetics in Medicine*.

